# CryoEM Visualization of an Adenovirus Capsid-Incorporated HIV Antigen

**DOI:** 10.1371/journal.pone.0049607

**Published:** 2012-11-14

**Authors:** Justin W. Flatt, Tara L. Fox, Natalia Makarova, Jerry L. Blackwell, Igor P. Dmitriev, Elena A. Kashentseva, David T. Curiel, Phoebe L. Stewart

**Affiliations:** 1 Department of Pharmacology and Cleveland Center for Membrane and Structural Biology, School of Medicine, Case Western Reserve University, Cleveland, Ohio, United States of America; 2 Department of Medicine, Division of Infectious Diseases, Emory Vaccine Center and Yerkes National Primate Research Center, Emory University, Atlanta, Georgia, United States of America; 3 Division of Cancer Biology, Department of Radiation Oncology, Washington University School of Medicine, St. Louis, Missouri, United States of America; French National Centre for Scientific Research, France

## Abstract

Adenoviral (Ad) vectors show promise as platforms for vaccine applications against infectious diseases including HIV. However, the requirements for eliciting protective neutralizing antibody and cellular immune responses against HIV remain a major challenge. In a novel approach to generate 2F5- and 4E10-like antibodies, we engineered an Ad vector with the HIV membrane proximal ectodomain region (MPER) epitope displayed on the hypervariable region 2 (HVR2) of the viral hexon capsid, instead of expressed as a transgene. The structure and flexibility of MPER epitopes, and the structural context of these epitopes within viral vectors, play important roles in the induced host immune responses. In this regard, understanding the critical factors for epitope presentation would facilitate optimization strategies for developing viral vaccine vectors. Therefore we undertook a cryoEM structural study of this Ad vector, which was previously shown to elicit MPER-specific humoral immune responses. A subnanometer resolution cryoEM structure was analyzed with guided molecular dynamics simulations. Due to the arrangement of hexons within the Ad capsid, there are twelve unique environments for the inserted peptide that lead to a variety of conformations for MPER, including individual α-helices, interacting α-helices, and partially extended forms. This finding is consistent with the known conformational flexibility of MPER. The presence of an extended form, or an induced extended form, is supported by interaction of this vector with the human HIV monoclonal antibody 2F5, which recognizes 14 extended amino acids within MPER. These results demonstrate that the Ad capsid influences epitope structure, flexibility and accessibility, all of which affect the host immune response. In summary, this cryoEM structural study provided a means to visualize an epitope presented on an engineered viral vector and suggested modifications for the next generation of Ad vectors with capsid-incorporated HIV epitopes.

## Introduction

Viruses and virus-like particles (VLPs) with capsid-incorporated or chemically-attached heterologous epitopes are being explored as vaccine platforms to provide protective immunity against pathogens [1]. The potential advantages of viral vaccine vectors include multivalent display of epitopes and the ability of viral particles to stimulate both the adaptive and innate immune systems. Recently it has been found that viral pathogens trigger innate immune sensors that recognize unique pathogen-associated molecular patterns (PAMPs) [2]. Vectors have been designed that display either antigenic peptides or, in some cases, whole protein domains. A hepatitis B VLP has been engineered to present a peptide from foot and mouth disease virus (FMDV), and this VLP elicits an immune response that is stronger than that from the FMDV peptide alone [3]. The hepatitis B VLP system has also been utilized to present GFP, and vectors have been produced that lead to a strong humoral immune response against GFP in rabbits [4]. In another example, a recombinant VLP was designed to display domains from the anthrax toxin receptor. A single administration of this VLP in rats led to a potent immune response against a lethal anthrax toxin challenge [5].

Adenoviral (Ad) based vectors have a number of advantages for vaccine applications including the ability to infect a broad range of target cells, the fact that their genome can accept large insertions of up to 8 kb, and their natural immunogenicity. In a recent study, a noninfectious, disrupted Ad vector with a covalently linked cocaine analog was found to evoke high-titer immunity to cocaine in mice [6]. Administration of this vaccine to mice was also found to reverse the hyperlocomotor activity induced by intravenous delivery of cocaine, suggesting that the vaccine-generated immune response blocks cocaine from reaching its target receptor in the brain. Ad vectors have also been explored for use in HIV vaccine approaches with antigens either expressed as a transgene or displayed on the capsid surface with an “antigen capsid-incorporation” strategy. A combination of these approaches was employed to generate a multivalent vaccine vector presenting an HIV antigen within the major surface exposed Ad capsid protein hexon [7]. Vectors were developed both with and without transgene expression of Gag. In this study, the capsid incorporated antigen was a twenty-four amino acid region of the HIV membrane proximal ectodomain region (MPER), derived from HIV glycoprotein gp41. This peptide region was selected for display on the Ad capsid because it is the target of one of the first identified broadly neutralizing anti-HIV-1 antibodies, 2F5 [8]. Vaccinations in mice with MPER incorporated Ad vectors elicited an HIV epitope-specific humoral immune response as well as an anti-HIV Gag cellular response [7].

The magnitude and quality of the immune responses generated by an engineered viral vector or designed immunogen is influenced by the structure and flexibility of the epitope, as well as the structural context and accessibility of the epitope [1]. Consideration of the structural environment of the epitope is particularly important in the case of viral based vaccines of the “capsid-incorporation” type where insertions can often be introduced at a variety of possible sites. Due to the spatial restrictions imposed by the packing of viral capsid proteins, different insertion sites are likely to tolerate various size insertions and lead to a range of surface exposure levels. Systematic investigations on the effect of the insertion point within the hepatitis B core antigen show that fusion to an immunodominant site in the middle of the nucleocapsid protein produces the strongest immune response for the inserted foreign epitope [9]. Furthermore, epitope fusions at the amino terminus of the nucleocapsid protein, which is not surface exposed in the VLP, correlated with the lowest level of immunogenicity.

Both the structure and flexibility of the presented epitope can affect the immune response. Neutralizing antibodies have been found for specific conformations, extended or α-helical, for neighboring residues within the MPER region. The broadly neutralizing monoclonal antibody 2F5 binds an extended conformation of MPER [10] while another such antibody, 4E10, targets an α-helical conformation [11]. An elegant paper by Ofek *et al*. [12] shows that by designing an immunogen with the proper conformation of MPER displayed on a protein scaffold it is possible to elicit antibodies that induce this region of gp41 to assume its 2F5-recognized shape. This paper also demonstrated a correlation between epitope flexibility and immunogenicity. Although all of the designed scaffold proteins presented similar extended conformations of MPER, the scaffolds with more flexibly held epitopes generated greater immune responses in animals. Antigen structure and flexibility has been shown to have an effect on antigen processing and presentation of helper T-cell epitopes [13]. The presence of flexible flanking regions around immunodominant helper T-cell epitopes against HIV gp120 is implicated in enhancing antigen processing in both mice and humans [13], [14].

Human clinical trials of candidate HIV vaccines have shown some modest efficacy, suggesting that it should be possible to generate vaccine-elicited protection against HIV-1 infection [15]. While induction of neutralizing antibodies is a major goal of HIV-1 vaccine development [15], an ideal vaccine would also induce cytotoxic T lymphocyte and helper T cell responses [14]. The field of rational vaccine design is still in the relatively early phase of figuring out the principles that correlate antigen display with the ultimate, multi-faceted immune response.

Atomic level structural information for the viral capsid is highly valuable during the vector design process. We reasoned that a structure of an engineered vector that produces an immune response would also be useful in the analysis phase and in guiding future vector design. In cases where the antigen is conformationally flexible, such as MPER of HIV gp41, it is not possible to know *a priori* which conformation will be presented on an engineered viral vector. We have undertaken a high resolution cryoEM structural study of the most immunologically promising Ad vector with a capsid-incorporated HIV MPER epitope described by Matthews *et al*. [7]. Our hypothesis is that an understanding of how the MPER epitope is presented on the viral capsid surface will lead to ideas for rationally modifying the next generation of Ad-based vectors to potentially produce a stronger immune response. This study illustrates that cryoEM combined with guided molecular dynamics simulations can be used to describe the structure and flexibility of an HIV epitope incorporated into an engineered Ad capsid.

## Materials and Methods

### CryoEM and Image Processing

A concentrated sample of the Ad-HVR2-GP41-L15 vector (∼0.2 mg/ml) was produced as described previously [7]. CryoEM samples were prepared with Quantifoil grids (Quantifoil Micro Tools GmbH) and a homebuilt vitrification device. An FEI Polara microscope (300 kV, FEG) operated at liquid nitrogen temperature was used for data acquisition. Digital micrographs were recorded on a Gatan UltraScan 4000 CCD camera at an absolute magnification of 397,878X, corresponding to a pixel size of 0.4 Å on the molecular scale. The defocus values of the micrographs ranged from −1 to −4 µm. Individual particles were selected from micrographs with in-house scripts that call IMAGIC subroutines [16] and computationally binned to generate matching particle image stacks with pixel sizes of 4.5 Å and 2.2 Å. The data with the coarser pixel size was used for the initial refinement rounds. The program CTFFIND3 [17] was used to determine initial estimates for the microscope defocus and astigmatism parameters. A cryoEM structure of Ad5.F35 [18] was used as the starting three-dimensional model for FREALIGN refinement [19]. The orientational and CTF parameters, as well as the absolute magnification values, were refined for the total dataset of 5,025 particle images. The resolution of the final reconstruction was estimated to be 8.7 Å by the Fourier Shell Correlation 0.5 threshold for the icosahedral capsid (radii 325–460 Å) (Fig. S1). Comparison of the cryoEM structure of Ad-HVR2-GP41-L15 with both the crystal structure of an intact Ad virion [20] and the cryoEM structure of Ad5.F35 [18] facilitated identification of density regions corresponding to the MPER insertions.

**Figure 1:**
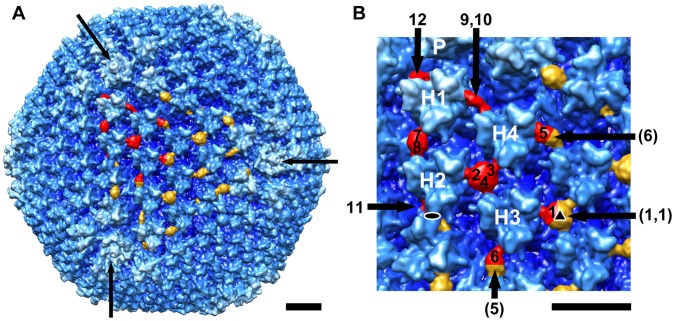
CryoEM structure of the Ad-HVR2-GP41-L15 vector at subnanometer resolution. (A) Full virion viewed along an icosahedral 3-fold axis. Density assigned to the MPER insertion within the top facet is colored in red and gold, with red representing the MPER density within one asymmetric unit. This Ad vector is based on human Ad type 5, which has long and flexible fibers (>300 Å). Only short portions of the fiber (out to a radius of 463 Å) have been reconstructed (3 fibers are indicated with arrows). (B) Enlarged view with the 12 MPER density regions within one asymmetric unit numbered 1–12. Interacting MPER density regions from adjacent asymmetric units are numbered in parentheses. The four hexons are labeled H1–H4 and the penton base is labeled P. The icosahedral 2- and 3-fold axes are indicated with oval and triangle symbols respectively. Scale bars represent 100 Å.

### CryoEM Guided Molecular Dynamics Simulations to Model the MPER Insertions

Given the diversity in sequence and structure for the gp41 MPER region in the PDB, we performed secondary structure prediction on the specific MPER sequence and neighboring linker residues that are incorporated into the Ad-HVR2-GP41-L15 vector (Fig. S2). The Jufo [21], SAM [22], and Psi-Pred [23] secondary structure methods were applied and the α-helical propensity results were averaged. Various initial models for the MPER section of the insertion were built with Swiss-PDB Viewer [24] including a long α-helix, and α-helices with a few extended residues at either end. Manual docking of these models into the cryoEM density aligned with the crystal structure of the intact Ad virion [20] led to selection of a model with four residues extended at the N-terminal end of the MPER sequence. An initial model for the 3-mer site was constructed using the selected monomeric model and the packing arrangement observed in the crystal structure of a partial MPER sequence stabilized with an isoleucine zipper motif (PDB-ID 3G9R) [25]. Additional 3-mer models were made with the individual helices rotated by +/−10°, 20°, 90°, and 180° along the helical axis to evaluate the packing interface.

Initial models for the 1-mer, 2-mer, and 3-mer MPER insertions were incorporated into hexon trimer coordinate files (PDB-ID 1VSZ) [20]. The molecular dynamics flexible fitting (MDFF) program [26] was used together with NAMD 2.8 [27] and VMD 1.9 [28] for cryoEM guided molecular dynamics simulations. All MDFF simulations were performed with implicit solvent, secondary structural restraints, a g-scale factor of 0.3, and the CHARMM force field. The 2-mer and 3-mer simulations involved extensive minimization (20,000 steps), followed by a 500 ps molecular dynamics phase, another minimization phase (40,000 steps), followed by a second 100 ps molecular dynamics phase, and final minimization (20,000 steps). The 1-mer models underwent less extensive simulations with initial minimization (20,000 steps), a 100 ps molecular dynamics phase, and final minimization (20,000 steps). Nonbonded and total potential energies were calculated using the NAMD Energy plugin. Simulated density maps were generated with the Molmap command in UCSF Chimera [29]. To simulate the 3-mer density from the final MDFF refined MPER coordinates, Molmap was run with three overlapping copies of the helical bundle related by 0°, 120° and 240° (Movie S1). All molecular graphics figures were made with UCSF Chimera. The MDFF simulations were run on Case Western Reserve University’s High Performance Computing Cluster.

**Figure 2:**
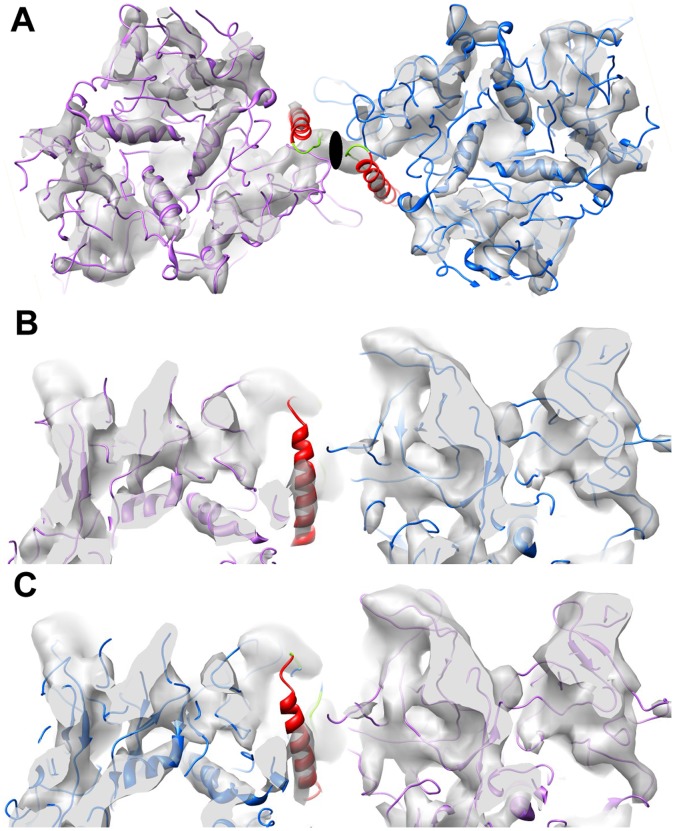
CryoEM density showing α-helices for two hexons and two MPER insertions. (A) Coordinates from the final frame of an MDFF simulation show α-helices of both hexon and MPER docked within density rods of the cryoEM structure (gray). (B) Perpendicular view showing one of the two MPER density rods. (C) Additional view showing the second of the two MPER density rods at the icosahedral 2-fold axis with the final MDFF coordinates, which were not icosahedrally constrained. The isosurface threshold levels were chosen to highlight the well resolved density rods within each panel. Ribbon representations are shown for the hexon backbone (purple and blue), the MPER sequence (red), and the linker regions (green). The icosahedral 2-fold axis is indicated with an oval symbol.

## Results

### CryoEM Structure of Ad Vector with Capsid Incorporated MPER Peptide

A recombinant type 5 adenovirus vector, Ad-HVR2-GP41-L15, was generated with a 24 amino acid MPER insertion within hypervariable region 2 (HVR2) of the hexon capsid protein [7]. The rationale for selecting a portion of MPER for incorporation was based on the fact that the gp41 envelope protein ectodomain is a target of three broadly neutralizing anti-HIV-1 antibodies [10]. The selection of the HVR2 insertion site within hexon was made after consideration of both the HVR2 and HVR5 sites, both of which were established as technically feasible capsid incorporation sites [30], [31]. HVR2 sites are immunogenic and near the top of the hexon trimer [32]. Based on atomic structural information for the intact Ad virion [20], [33], we reasoned during the design phase that insertions at this site should be exposed on the viral capsid surface. A purified sample of Ad-HVR2-GP41-L15 was preserved on cryoEM grids and imaged with an FEI Polara microscope (300 kV, FEG). Over 5,000 cryoEM particle images were processed to generate a structure at 9 Å resolution (FSC 0.5) for the icosahedral capsid (Fig. 1A). MPER density is observed between adjacent hexons in the capsid. The size and shape of the MPER density varies as seen in the asymmetric unit of the capsid (Fig. 1B). There are four hexon trimers in the asymmetric unit (H1-H4) displaying in total 12 MPER insertions, each with a unique environment within the capsid.

**Figure 3:**
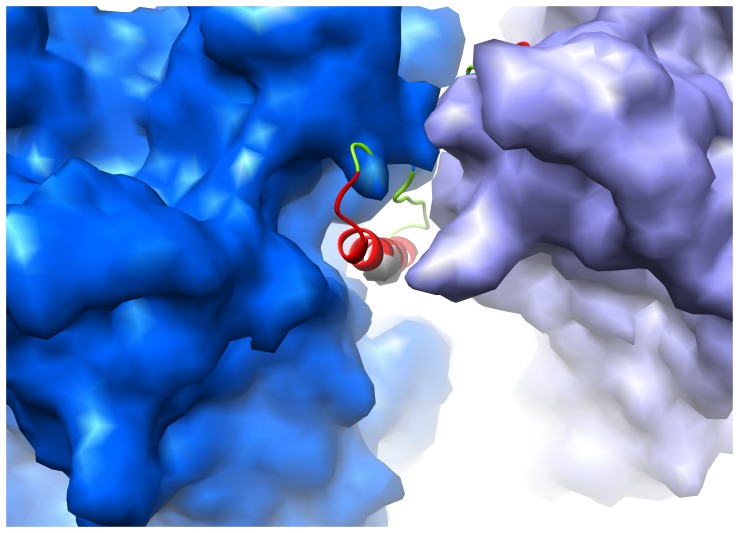
MPER insertion within a narrow cavity between hexons at the icosahedral 2-fold axis. Density for two hexons (blue and purple) simulated from the final MDFF frame shown surrounding experimentally determined cryoEM density (gray) for one MPER insertion. A ribbon representation of the final MDFF model of the MPER insertion is overlaid, colored as in Figure 2. A tilted view is shown to emphasize the confined nature of the cavity between the hexons.

**Figure 4:**
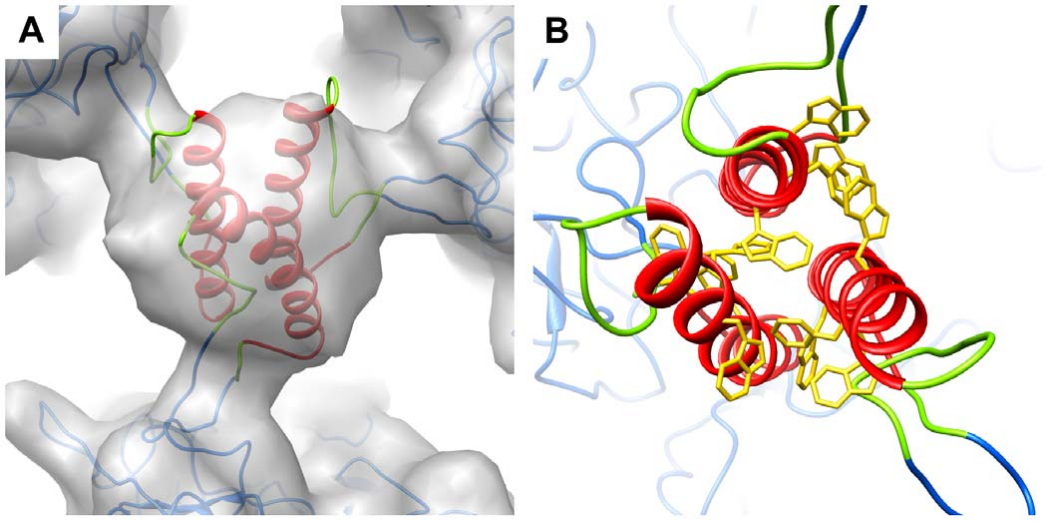
MPER forms a stable helical bundle at 3-mer sites. (A) Overlay of MDFF refined model (ribbons) and the cryoEM density (gray) at the icosahedral 3-fold axis. The ribbon coloring scheme is the same as in Figure 2. (B) Enlarged view of the helical packing interface viewed along the bundle axis with aromatic sidechains displayed (gold).

At the icosahedral 3-fold axis, MPER insertions from three neighboring hexons meet and form one merged density region. A similar 3-way interaction between MPER insertions is observed at a position of local 3-fold symmetry between hexons H2, H3 and H4. Smaller regions of density are also observed between pairs of hexons, where two MPER insertions are displayed in close proximity (Fig. 1B). These 3-mer and 2-mer MPER interactions account for 10 of the 12 insertion sites within the asymmetric unit. At the remaining two sites, which are near the icosahedral 2-fold axis and near the penton base, density is observed for isolated, non-interacting (1-mer) MPER peptides.

**Figure 5:**
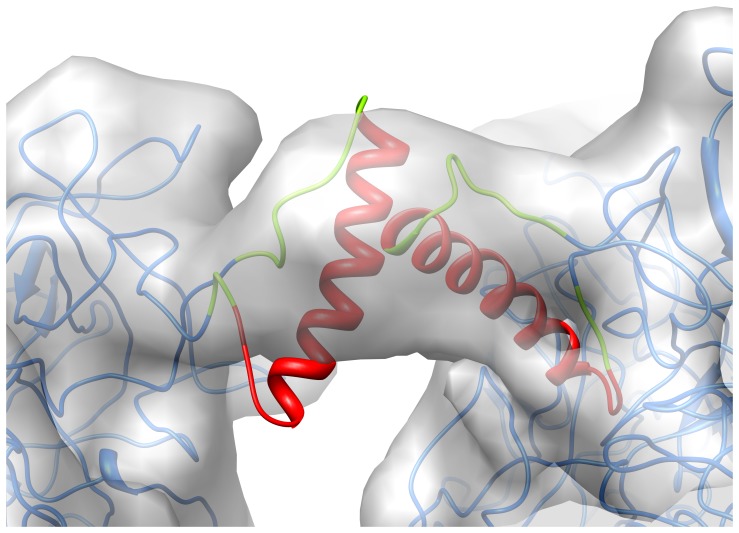
MPER interactions at 2-mer sites are weak and transient. Overlay of MDFF refined model (ribbons) and the cryoEM density (gray) at the strongest 2-mer site (between hexons H3 and H4 of a neighboring asymmetric unit). The ribbon coloring scheme is the same as in Figure 2.

### Alpha-helices are Observed within the Ad Capsid and for one MPER Insertion

In subnanometer (<10 Å) resolution cryoEM structures, well-ordered α-helices should be resolved as density rods. Indeed, α-helices within hexon trimers are clearly resolved as rods within the cryoEM structure of Ad-HVR2-GP41-L15 (Fig. 2). The MPER epitope is known to adopt numerous conformations, some α-helical and some extended, and its structural plasticity may be important in facilitating viral membrane fusion. A crystal structure of the MPER region stabilized with an isoleucine zipper motif reveals a parallel triple-stranded coiled coil [25]. An NMR structure of an MPER segment in the presence of DPC micelles shows a kinked conformation with two separate helical segments [34]. We observe a well resolved density rod, indicative of an α-helix, at the 1-mer site near the icosahedral 2-fold axis of the capsid (Fig. 2). At the other 1-mer site near the penton base, some density for MPER is observed along the side of hexon, but there is not a clearly resolved α-helix. The strength of the cryoEM density varies among the 2-mer and 3-mer sites, indicating different levels of conformational heterogeneity, but none of these regions have resolved α-helices.

**Figure 6:**
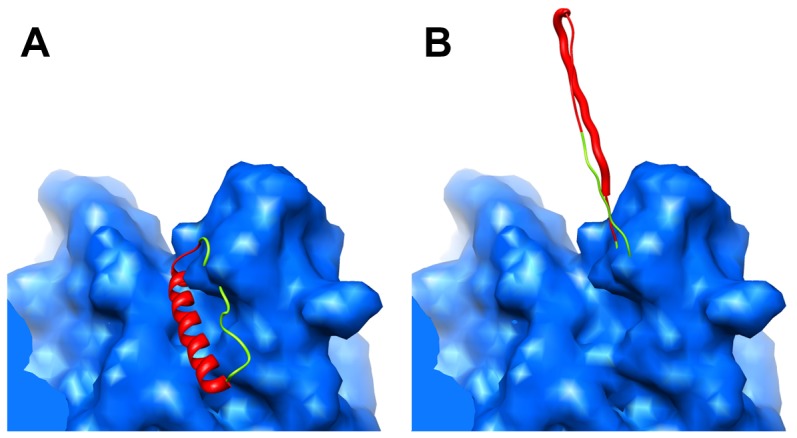
Alternate model conformations for MPER next to penton base. (A) Simulated density for hexon (blue) shown with a ribbon representation of an MDFF model of the MPER in an α-helical conformation or (B) extended conformation. The 14 amino acid 2F5 epitope is shown with a thicker ribbon in the extended model. The ribbons are colored as in Figure 2.

**Figure 7:**
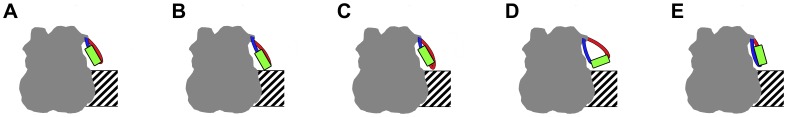
Proposed vector modifications for optimizing MPER presentation at the Ad hexon HVR2 site. (A) Schematic representation of the MPER insertion incorporated within the Ad-HVR2-GP41-L15 vector. The hexon capsid protein (gray) is shown together with the N-terminal 3aa linker (blue), the MPER peptide (green), and the C-terminal 10aa linker (red). The protein-protein interface between hexons in the Ad capsid is represented by a hashed region. (B-E) Based on the structural analysis of the Ad-HVR2-GP41-L15 vector, four possible modifications are proposed which include (B) extending the N-terminal linker by 3aa, (C) extending the C-terminal linker by 4aa, (D) extending both the N- and C-terminal linkers by 2aa, and (E) swapping the N- and C- terminal linkers.

### MPER Conformation is Constrained by the Ad Capsid at One Insertion Site

The variation in the MPER cryoEM density at the 12 unique insertion sites is a reflection of diverse local environments created by the hexon and penton base packing arrangement within the Ad capsid. At the icosahedral 2-fold axis, hexon packing restricts the MPER insertion to within a narrow cavity (Fig. 3). The cryoEM structure shows a well resolved density rod, which suggests that the insertion at this site adopts a rigid helical structure with limited flexibility. Interactions with other MPER insertions are precluded at this site. To further interpret the structure, we used cryoEM guided molecular dynamics simulations. We built initial atomic models based on the available MPER structures and secondary structure prediction for the inserted sequence (Fig. S2). These models served as input for Molecular Dynamics Flexible Fitting (MDFF) simulations [26]. The MDFF simulation for the 1-mer site near the icosahedral 2-fold axis reveals that an α-helix for the majority of the 24 amino acid MPER sequence fits within the narrow cavity between hexons.

### Strong Helical Interactions between MPER Insertions at the 3-mer Sites

Analysis of the cryoEM density at the 3-mer positions indicates that a stable interaction between MPER insertions occurs as the observed MPER density is as strong as the hexon density in the Ad capsid. Based on the crystal structure of the MPER region stabilized with an isoleucine zipper motif [25], we speculated that 3 nearby MPER insertions might interact as a parallel triple-stranded coiled coil. To test this idea, we built an atomic model for three interacting MPER α-helices based on the parallel coiled coil crystal structure [25]. MDFF simulations were performed with the atomic model docked into cryoEM density. Various initial helical interfaces (with +/−10°, 20°, 90°, and 180° rotations about the helical axis) were tested to optimize the helical packing arrangement. The selected final model for the triple helical bundle has a minimum in both the Nonbonded and Total Potential energy terms calculated between the helical MPER residues (Table S1). The MDFF refined model for the MPER interaction at the icosahedral 3-fold axis fits within the cryoEM density and displays a reasonable sidechain packing arrangement (Fig. 4). This model fits equally well into the density at the other unique 3-mer site within the Ad capsid (between hexons H2, H3 and H4). Simulated density based on the MDFF refined model closely resembles the cryoEM density after 3-fold averaging (Fig. S3, Movie S1). Modeling indicates that the length and flexibility of the linker regions of the MPER insertion would allow the triple helical bundle to tilt in various directions. This would result in an average representation in the cryoEM structure and explain why resolved density rods for α-helices are not observed at the 3-mer sites.

### Transient Interactions between MPER Insertions at the 2-mer Sites

The cryoEM density at the three 2-mer sites within the asymmetric unit is variable with the strongest density region between hexons H3 and H4 of a neighboring asymmetric unit (Fig. 1B). All of the 2-mer density regions are weaker than those at the 3-mer sites. We built an initial atomic model for two interacting MPER α-helices based on the MDFF refined 3-mer model. However, during MDFF simulations the two helices drifted apart and ended up as only weakly interacting (Fig. 5). Various starting models docked within the strongest cryoEM 2-mer density were tested in MDFF, but the final Nonbonded and Total Potential energy terms were not as favorable as for the 3-mer model. The density differences observed between the three unique 2-mer sites in the cryoEM structure can be explained by variation in distances between the insertion sites of neighboring hexons (Table S2). The closer the insertion sites are, the weaker the cryoEM density is in that region. This implies that if the insertion sites are too close then favorable interactions between neighboring MPER sequences are less likely to be formed. Taken together, the cryoEM and modeling results indicate that the MPER interactions at 2-mer sites are transient and weak in nature.

### Conformational Flexibility of MPER Next to Penton Base

The MPER insertion closest to the penton base has a unique local environment within the capsid. We observe a small amount of MPER density for this insertion along the side of the peripentonal hexon facing the penton base. However, the density is not well defined, indicating a low affinity binding site for MPER on the side of the hexon. Therefore, we speculate that the MPER insertion at this site is the most conformationally flexible within the Ad capsid.

## Discussion

A hybrid cryoEM and molecular dynamics approach enabled us to model the conformation of MPER presented on an engineered Ad vaccine vector that shows a promising immunological response in mice [7]. There are twelve unique hexon HVR2 insertion sites within the Ad capsid that result in different MPER conformations, including well resolved individual α-helices at the icosahedral 2-fold axis, interacting α-helices, and partially extended forms. At the insertion site closest to the icosahedral 2-fold axis, a well resolved density rod is observed for MPER, strongly suggesting that the MPER sequence is adopting an α-helical conformation at this position in the capsid. This MPER insertion site is constrained by the Ad capsid with the helix observed within a narrow cavity between two hexons. We speculate that the MPER sequence presented at this confined site on the Ad capsid would not be accessible to antibody molecules. The observation of strong density at the 3-mer sites indicates a stable interaction between MPER regions at these capsid locations. MDFF simulations with atomic models based on a crystal structure of a partial MPER sequence that forms a parallel triple helical bundle reveal that a favorable helical packing arrangement can be achieved for the MPER sequence in the Ad-HVR2-GP41-L15 vector. The MDFF refined 3-mer model has the aromatic sidechains buried at the helical interfaces. The cryoEM density at the 3-mer sites can be reasonably well simulated by 3-fold averaging the final helical bundle model. Given the strength of the cryoEM density at the 3-mer sites it seems likely that these MPER regions are stably associating and thus are unlikely to be available for interaction with antibodies.

In comparison, the cryoEM density at the 2-mer sites is more variable. MDFF simulations show a more limited interaction between MPER regions where only two insertions can interact. The 1-mer insertion site next to the penton base leads to little observable cryoEM density for the MPER region. Although we cannot directly observe flexibility of a peptide region in a cryoEM structure, the presence of small amino acids (Gly and Ser) in the linkers, and the weak nature of the density, both suggest flexible MPER presentation at these sites. The 2-mer insertions could be in equilibrium between interacting with a neighboring insertion and forming an extended and flexible conformation above the capsid. Similarly, the 1-mer sites next to the penton base could be in equilibrium between interacting with hexon and forming an extended conformation (Fig. 6). The presence of extended MPER conformations on the Ad-HVR2-GP41-L15 vector is supported by the observation that this vector binds the human HIV monoclonal antibody 2F5 [7], which recognizes an extended 14 amino acid region of MPER.

A major obstacle to the rational design of viruses as vaccine vectors using the capsid-incorporation strategy is the lack of understanding of the rules governing the relationship between epitope presentation and host immune response. We reasoned that a structural analysis of the Ad-HVR2-GP41-L15 vector would lead to ideas on how to modify the next generation of Ad-based vectors and potentially produce stronger immune responses. Consideration of the modeled MPER structure at the most constrained position within the Ad capsid led us to propose several modifications that should be structurally tolerated at the hexon HVR2 site (Fig. 7). We were careful to avoid increasing the insertion length so much that it might interfere with hexon-hexon packing interactions during capsid assembly. One possible modification would be to add a maximum of 3aa to the N-terminal linker, while keeping the MPER insertion sequence and the C-terminal linker the same (Fig. 7B). Modeling indicates that this longer insertion would still fit at the constrained 1-mer location within the capsid, while enhancing the flexibility of the MPER insertions at the 1-mer site next to the penton base and at the 2-mer sites. A second possibility is to increase the C-terminal linker by up to 4aa (Fig. 7C). Alternatively, both the N- and C-terminal linkers could potentially be lengthened by 2aa (Fig. 7D). All three of these proposed modifications should enhance the exposure of the heterologous MPER epitope on the capsid surface and potentially enhance the generation of antibodies. In addition, the lengthened, flexible linkers could improve antigen processing and subsequent presentation on helper T-cells [14]. The fourth proposed vector modification is a swap of the N- and C-terminal linkers (Fig. 7E), which modeling indicates would be structurally permissible at all 12 insertion sites within the asymmetric unit. This would put the longer, and presumably more flexible, linker before the MPER epitope and would subtly alter antigen presentation on the capsid as well as potentially enhance antigen processing [13], [14].

The structural information gained from this study could be applicable to the design of vectors with alternative HIV epitopes within the Ad hexon HVR2 site. More than a dozen broadly neutralizing anti-HIV monoclonal antibodies have been isolated [15]. Once they are structurally characterized in complex with their cognate epitopes, this information could be used to design the optimal epitope insertion, similar in length to the MPER α-helix, for display at the Ad hexon HVR2 sites. Moreover, future studies are in development to test which of the predicted modifications will lead to increased immunogenicity. In summary, this cryoEM study provided a way to visualize and model the presentation of a heterologous epitope incorporated within an Ad capsid and suggested potential ways to optimize MPER presentation on the Ad capsid.

## Supporting Information

Figure S1 **Resolution assessment of the Ad-HVR2-GP41-L15 cryoEM structure.** The Fourier shell correlation (FSC) curve is calculated for the icosahedral capsid (radii 325–460 Å). The resolution as assessed by the FSC = 0.5 criterion is 8.7 Å.
(TIF)

Figure S2 **Secondary structure prediction for the inserted MPER and linker sequence.** Average of the predicted α-helical propensity as a function of the amino acid sequence, including the 24aa MPER and the N- and C-terminal linkers. The prediction is an average of the results from Jufo, SAM, and Psi-Pred. The linkers are shown in green, and the MPER sequence is shown in purple for the region modeled as extended, and red for the region modeled as α-helical. The MPER and linker sequence was inserted within the hexon HVR2 regions after Val-188 and before Pro-193.
(TIF)

Figure S3 **Comparison of cryoEM density at the icosahedral 3-fold axis with simulated hexon/MPER density.** (A) Top view of the cryoEM density contoured to show the MPER insertion density between three hexons. (B) Corresponding view of the simulated density for three hexons (blue) and the MPER residues of the MDFF refined 3-mer model (red). The simulated hexon density is filtered to 8 Å resolution. The simulated MPER density is filtered to 12 Å resolution and 3-fold averaged to account for the observation that the flexible linkers would presumably allow the helical bundle to tilt in three different directions. Ribbon representations are shown for the hexon backbone (blue), the MPER sequence (red), and the linker regions (green). (C and D) Perpendicular views.
(TIF)

Table S1 **Optimization of the helical interface at a 3-mer site with molecular dynamics flexible fitting.**
(DOCX)

Table S2 **Distances between hexon insertion sites at 2-mer sites.**
(DOCX)

Movie S1 **Simulated density for the MPER 3-mer insertion.** The movie starts with the cryoEM density (gray) of the 3-mer MPER between three hexons at the icosahedral 3-fold axis of the Ad-HVR2-GP41-L15 structure. The hexon coordinates (blue ribbons) are docked into the density, followed by the MDFF refined 3-mer model (red ribbons). Three copies of the MPER model are shown sequentially (rotated 120° with respect to each other). Simulating density from these three MPER coordinate sets leads to 3-fold averaged MPER density (red), which is shown with various isosurface levels until it approximates the experimental cryoEM density.
(MOV)
