# Supplementary figures and images for: CryoEM Visualization of an Adenovirus Capsid-Incorporated HIV Antigen
Source: PLoS One. 2012 Nov 14;7(11):e49607. doi: 10.1371/journal.pone.0049607 (PMC3498208; doi:10.1371/journal.pone.0049607)

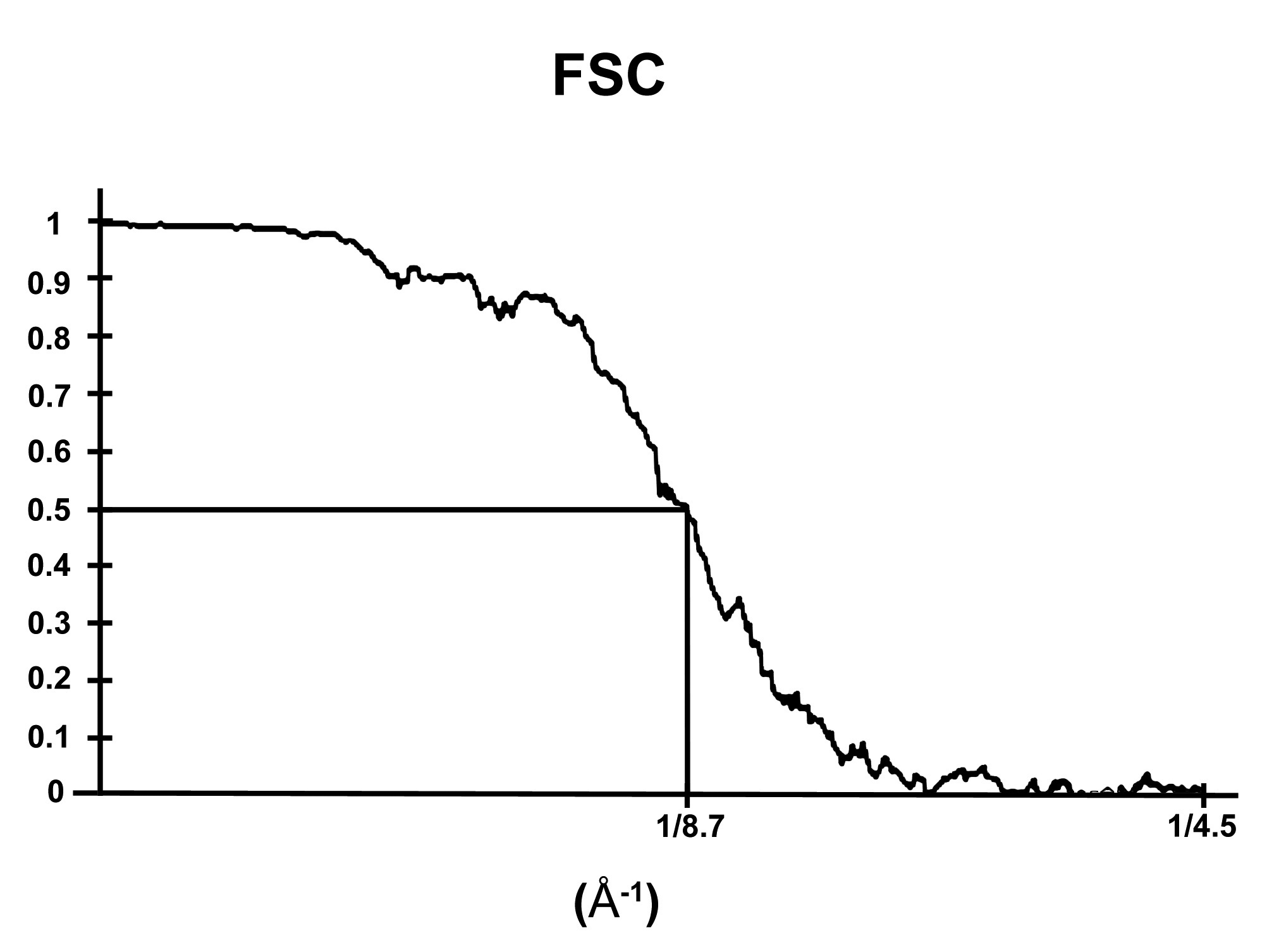

Supplement: Figure S1 — Resolution assessment of the Ad-HVR2-GP41-L15 cryoEM structure. The Fourier shell correlation (FSC) curve is calculated for the icosahedral capsid (radii 325–460 Å). The resolution as assessed by the FSC = 0.5 criterion is 8.7 Å. (TIF) [file pone.0049607.s001.tif]

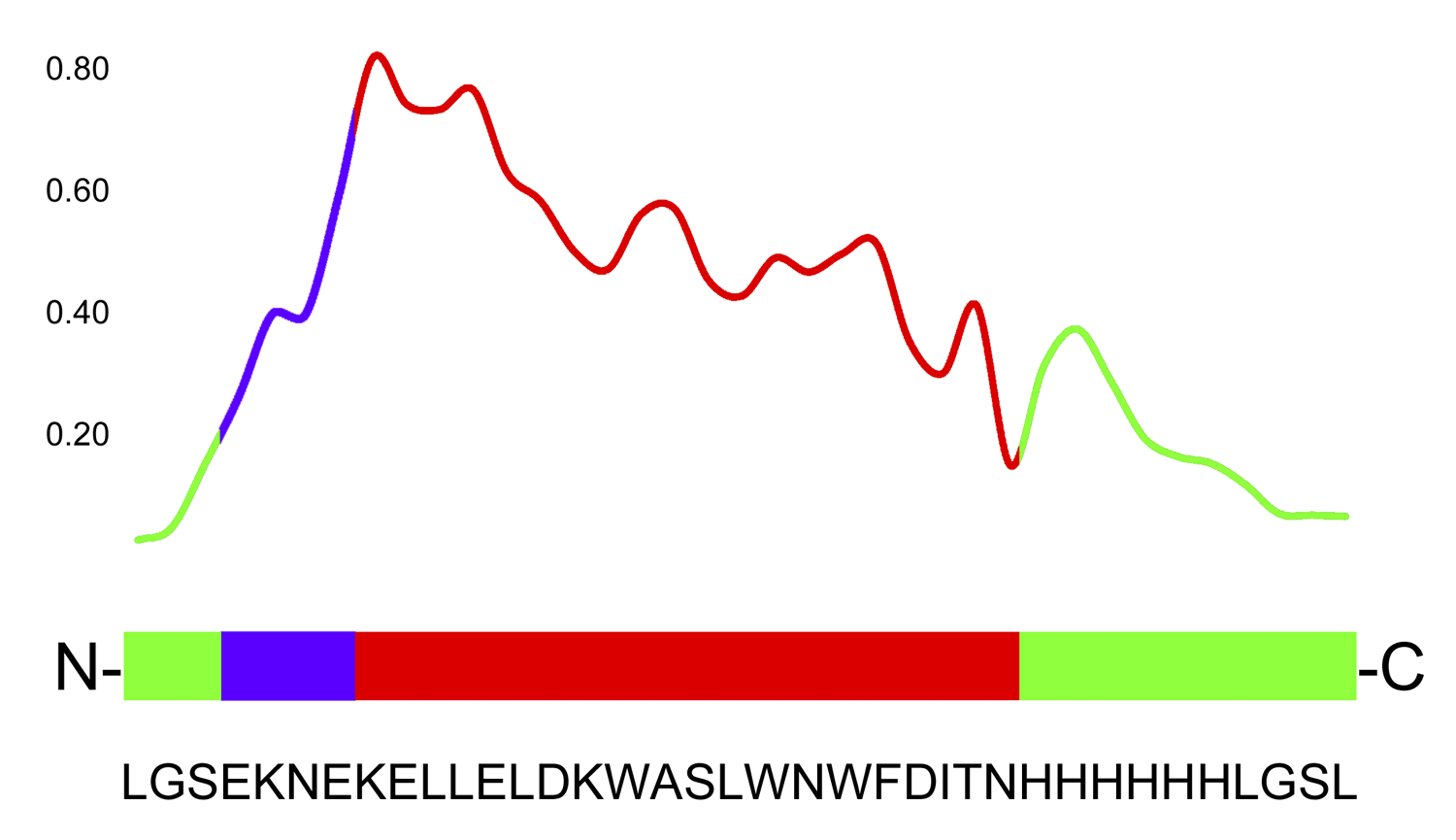

Supplement: Figure S2 — Secondary structure prediction for the inserted MPER and linker sequence. Average of the predicted α-helical propensity as a function of the amino acid sequence, including the 24aa MPER and the N- and C-terminal linkers. The prediction is an average of the results from Jufo, SAM, and Psi-Pred. The linkers are shown in green, and the MPER sequence is shown in purple for the region modeled as extended, and red for the region modeled as α-helical. The MPER and linker sequence was inserted within the hexon HVR2 regions after Val-188 and before Pro-193. (TIF) [file pone.0049607.s002.tif]

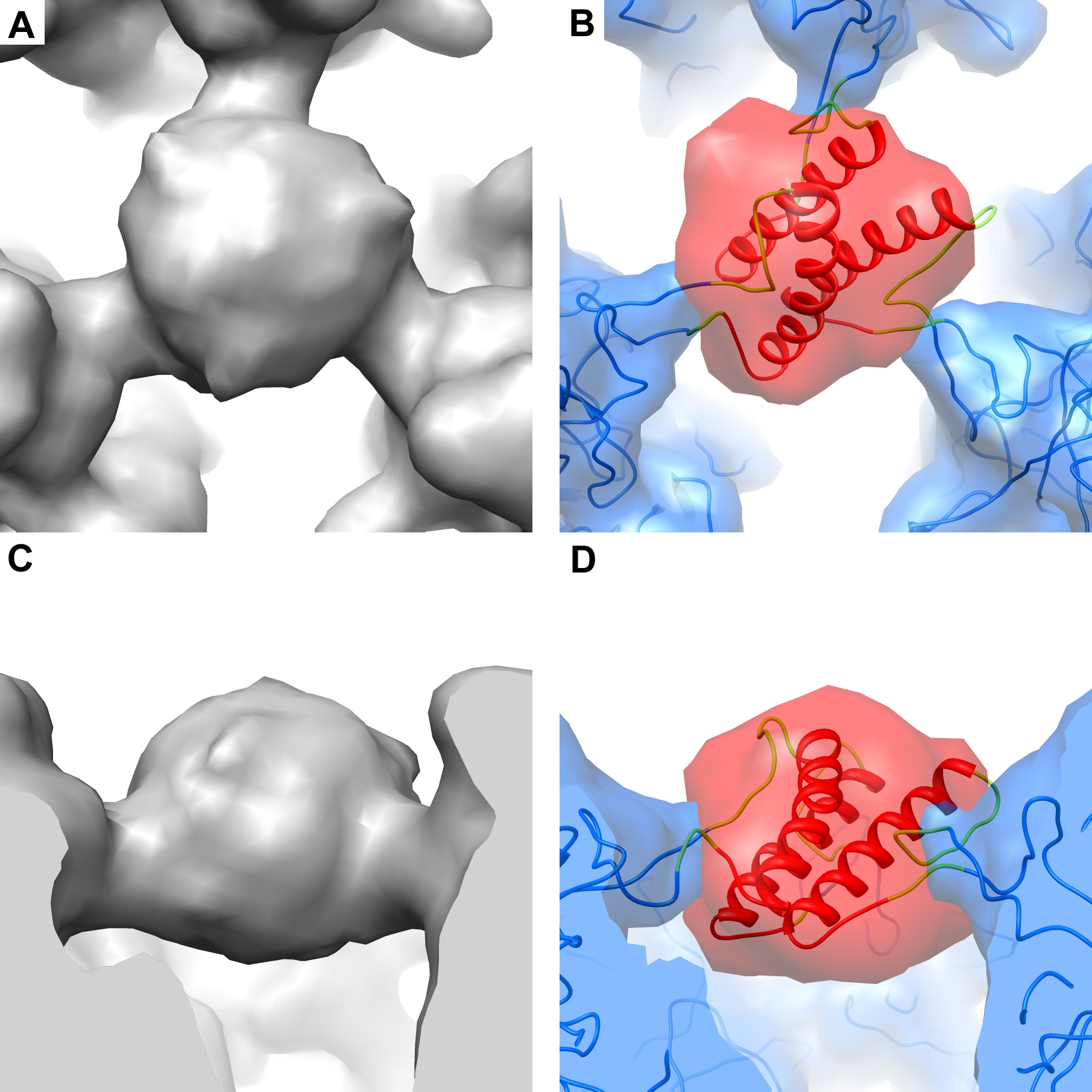

Supplement: Figure S3 — Comparison of cryoEM density at the icosahedral 3-fold axis with simulated hexon/MPER density. (A) Top view of the cryoEM density contoured to show the MPER insertion density between three hexons. (B) Corresponding view of the simulated density for three hexons (blue) and the MPER residues of the MDFF refined 3-mer model (red). The simulated hexon density is filtered to 8 Å resolution. The simulated MPER density is filtered to 12 Å resolution and 3-fold averaged to account for the observation that the flexible linkers would presumably allow the helical bundle to tilt in three different directions. Ribbon representations are shown for the hexon backbone (blue), the MPER sequence (red), and the linker regions (green). (C and D) Perpendicular views. (TIF) [file pone.0049607.s003.tif]
