# Supplementary material for: CryoEM Visualization of an Adenovirus Capsid-Incorporated HIV Antigen
Source: PLoS One. 2012 Nov 14;7(11):e49607. doi: 10.1371/journal.pone.0049607 (PMC3498208; doi:10.1371/journal.pone.0049607)
Supplement: Table S1 — Optimization of the helical interface at a 3-mer site with molecular dynamics flexible fitting. (DOCX) [file pone.0049607.s004.docx]

| **Table S1. Optimization of the helical interface at a 3-mer site with molecular dynamics flexible fitting.^1,2^** | | | | | | | | |
| --- | --- | --- | --- | --- | --- | --- | --- | --- |
|  |  |  |  |  |  |  |  |  |
|  | **-90°** | **-20°** | **-10°** | **minimum** | **+10°** | **+20°** | **+90°** | **+180°** |
| Nonbonded | -832.4 | -667.6 | -876.9 | -922.1 | -680.5 | -837.6 | -447.5 | -528.4 |
| Total Potential | -510.1 | 547.2 | -567.9 | -626.5 | -383.2 | -565.1 | -146.7 | -207.7 |
|  |  |  |  |  |  |  |  |  |
| ^1^Energies reported are between helical MPER residues in kcal/mol | | | | | | | | |
| ^2^Rotation of individual helices along their helical axes with respect to the initial model | | | | | | | | |
