# Supplementary material for: CryoEM Visualization of an Adenovirus Capsid-Incorporated HIV Antigen
Source: PLoS One. 2012 Nov 14;7(11):e49607. doi: 10.1371/journal.pone.0049607 (PMC3498208; doi:10.1371/journal.pone.0049607)
Supplement: Table S2 — Distances between hexon insertion sites at 2-mer sites. (DOCX) [file pone.0049607.s005.docx]

| **Table S2. Distances between hexon insertion sites at 2-mer sites.^1^** | | |
| --- | --- | --- |
|  |  |  |
| **Location of 2-mer** | **Distance (A)** | **Relative strength of cryoEM density** |
| Between Hexons 3 and 4^2^ | 34 | Strong |
| Between Hexons 1 and 2 | 29 | Intermediate |
| Between Hexons 1 and 4 | 28 | Weak |
|  |  |  |
| ^1^Average of measured distances between pairs of Val-188 and Pro-193 hexon residues | | |
| ^2^Hexon 4 is of a neighboring asymmetric unit | | |
